# A Novel Missense SNRNP200 Mutation Associated with Autosomal Dominant Retinitis Pigmentosa in a Chinese Family

**DOI:** 10.1371/journal.pone.0045464

**Published:** 2012-09-19

**Authors:** Tiecheng Liu, Xin Jin, Xuemin Zhang, Huijun Yuan, Jing Cheng, Janet Lee, Baoquan Zhang, Maonian Zhang, Jing Wu, Lijuan Wang, Geng Tian, Weifeng Wang

**Affiliations:** 1 Department of Ophthalmology, Chinese PLA General Hospital, Beijing, China; 2 Department of Ophthalmology, Hainan Branch of Chinese PLA General Hospital, Sanya, China; 3 Institute of Otolaryngology, Chinese PLA General Hospital, Beijing, China; 4 Department of Ophthalmology and Shiley Eye Center, University of California San Diego, La Jolla, California, United States of America; 5 Department of Science and Technology, BGI-Tianjin, Tianjin, China; 6 Department of Reproductive Health, BGI-Shenzhen, Shenzhen, China; 7 Department of Gastroenterology and Hepatology, Hainan Branch of Chinese PLA General Hospital, Sanya, China; Lousiana State University Health Sciences Center, United States of America

## Abstract

The *SNRNP200* gene encodes hBrr2, a helicase essential for pre-mRNA splicing. Six mutations in *SNRNP200* have recently been discovered to be associated with autosomal dominant retinitis pigmentosa (adRP). In this work, we analyzed a Chinese family with adRP and identified a novel missense mutation in *SNRNP200*. To identify the genetic defect in this family, exome of the proband was captured and sequencing analysis was performed to exclude known genetic defects and find possible pathogenic mutations. Subsequently, candidate mutations were validated in affected family members using Sanger sequencing. A novel missense mutation, c.2653C>G transition (p.Q885E), in exon 20 of *SNRNP200* was identified. The mutation co-segregated with the disease phenotype over four generations and was absent in 100 normal unaffected individuals. This mutation occurs at highly conserved position in hBrr2 and is predicted to have a functional impact, suggesting that hBrr2-dependent small nuclear riboproteins (snRNPs) unwinding and spliceosome activation is important in the pathogenesis of some variants of RP.

## Introduction

Retinitis pigmentosa (RP; OMIM 268000) is a group of inherited retinal dystrophies characterized by the progressive degeneration of photoreceptors. RP typically begins with night blindness in the early teenage years followed by decreasing visual fields, leading to tunnel vision and eventually legal blindness [1]. Fundus examination findings include bone-spicule pigmentation, attenuation of the retinal blood vessels, and waxy pallor of the optic disk. Electroretinography (ERG) measures the electrical response of the retina and is the most reliable diagnostic tool for RP [2].

The worldwide prevalence of RP is approximately 1 in 3,000 to 5,000 individuals [3]. Inheritance can be autosomal dominant, autosomal recessive, and X-linked. Digenic and mitochondrial inheritance is rare but has also been described [4]. The occurrence of autosomal dominant RP (adRP) varies among different ethnic groups, with an estimated average frequency of 30–40% of RP cases [5]. To date, mutations in eighteen genes have been associated with adRP (RetNet: http://www.sph.uth.tmc.edu/retnet/sum-dis.htm, Last updated May 4, 2012), of which five genes had been found in Chinese adRP patients [6]–[9].

*SNRNP200* was firstly identified as an adRP pathogenic gene in two Chinese families by linkage scan of genomic regions containing known candidate genes in 2009 [10]. Currently, there has been six mutations reported in *SNRNP200,* of which p.S1087L and p.R1090 [10]–[11] mutations were considered as the pathogenic genes of the Chinese adRP families and the other four mutations, p.R681C, p.R681H, p.V683L and p.Y689C, were found in a cohort of 96 North American patients with adRP [12].

We report a novel missense mutation in *SNRNP200* in a Chinese family with adRP. This mutation was identified using a combined approach of exome sequencing and candidate mutation validation.

## Methods

### Participants and Clinical Data

**Figure 1:**
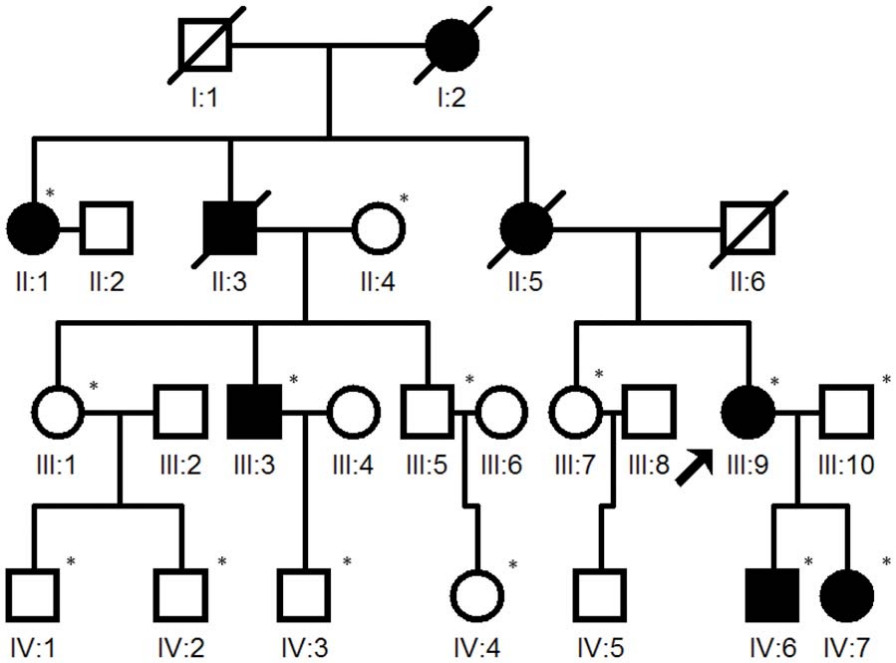
Pedigree of the adRP family. Squares and circles indicate males and females respectively, darkened symbols represent affected members, and slashed symbols denote that the subject is deceased. The arrow points to the proband. An asterisk indicates the subject underwent clinical and genetic analyses.

The research received approval from the Ethic Committee of Chinese PLA General Hospital and was carried out in accordance with the Declaration of Helsinki. Written informed consent was obtained from all participants prior to participation in the study. A four-generation Chinese family (Figure 1) from Sichuan province in China diagnosed with retinitis pigmentosa was identified and followed-up clinically at the Chinese PLA General Hospital. There are 24 members in this four-generation Chinese family and 14 individuals were recruited for the study, including 5 affected individuals and 9 unaffected individuals of which two were unrelated spouses. All participants underwent a full medical history and comprehensive ophthalmic evaluation, including best correct visual acuity, slit lamp examinations, applanation tonometry, direct funduscopy, visual field test and ERG. The study also included 100 normal controls that were randomly collected in outpatient department of Chinese PLA General Hospital. The controls had no symptoms of night blindness,vision reduction and any personal or family history of known inheritance disease. All underwent detailed ophthalmic examinations such as visual acuity, intraocular pressure, slit lamp, fundus examination, visual field test and ERG.

### Exon Capture and Next-generation Sequencing

Genomic DNA was isolated from peripheral leukocytes using the TIANamp Blood DNA Kit (Tiangen Biotech Co. Ltd, Beijing, China). The exome sequencing approach was used to identify the disease-causing genetic variants for the adRP family in this study. 15 µg of genomic DNA sample from the proband (Figure 1, III: 9) was randomly fragmented by Covaris and the size distribution of the resulting fragments was between 250 bp and 300 bp. Adapters were ligated to the genomic fragments, and the ligated fragments were amplified by ligation-mediated PCR (LM-PCR). The products were purified and hybridized to the NimbleGen SeqCap EZ Library for enrichment; non-hybridized fragments were washed out. Both non-captured and captured LM-PCR products were subjected to quantitative PCR to estimate the magnitude of enrichment. Each captured library was then loaded on the Hiseq2000 platform, and high-throughput sequencing was performed for each captured library to ensure that each sample met the desired average sequencing depth. Raw image files were processed by Illumina basecalling Software 1.7 for base-calling with default parameters and the sequences of each individual were generated as 90 bp pair-end reads. Detailed information on the NimbleGen SeqCap EZ Library can be found at http://www.nimblegen.com/products/seqcap/ez/index.html.

### Read Mapping and Variant Analysis

The human reference genome (NCBI build 37.1) and its gene annotation were downloaded from the UCSC database (http://hgdownload.cse.ucsc.edu/goldenPath/hg19/bigZips/). Reads were mapped onto the reference sequences using SOAPaligner/SOAP2 for SNP analysis and BWA (http://bio-bwa.sourceforge.net/) for Indel analysis. Only mapped reads were used for subsequent analysis. Coverage and depth calculations were based on all mapped reads and the exome region. All changes from the proband were filtered against exome data from dbSNP132 (http://hgdownload.cse.ucsc.edu/goldenPath/hg19/database/snp132.txt.gz.), HapMap project (ftp://ftp.ncbi.nlm.nih.gov/hapmap), 1000 Genome Project (ftp://ftp.1000genomes.ebi.ac.uk/vol1/ftp) and YH database (http://yh.genomics.org.cn/).

### Mutation Validation

After filtering against multiple databases, Sanger sequencing was used to determine if any of the potential novel mutations that were in known causative genes of RP co-segregated with the disease phenotype in this adRP family. The mutations were then confirmed in all 14 family members by Sanger sequencing. Direct polymerase chain reaction (PCR) products were sequenced using Bigdye terminator v3.1 cycle sequencing kits (ABI, Foster City, CA, USA) and analyzed on an ABI 3700XL Genetic Analyzer. The primers are listed in Table 1.

**Table 1:** Primers used for potential mutations amplification.

Mutation	Gene	Forward primer(5′-3′)	Reverse primer(5′-3′)	Product length(bp)
2653C>G	*SNRNP200*	CCTGCATCAAAATTCAGACA	ACAATAGGGACCGACCCACT	329
1415G>A	*PDE6B*	ACAGGTGGGAAAGTCAGCAG	CACTGGCTGAGACTGAAAGC	457
10246T>G	*USH2A*	ATCTGCATTTTCAGCAGCTT	CTTCCTAGTATACAAACTGCCTTCA	441
2750G>A	*USH2A*	CCGATCGGCTGAGTTTTATC	CTTTCCTCTGTCTGCCTTGC	517
997T>C	*USH2A*	GGCATTTGTTGCAATAACCA	TGAAAGCACTAAACGAGTGACA	450

## Results

### Clinical Findings

**Figure 2:**
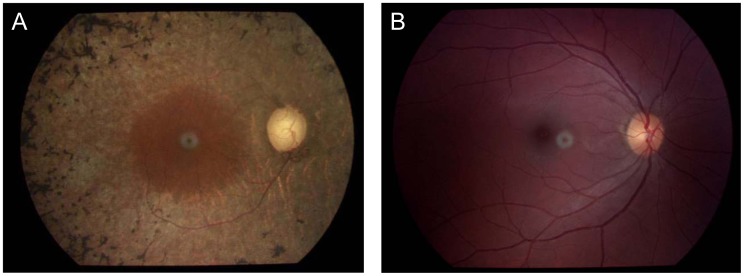
Fundus photography of the proband and a normal subject. (A) Attenuation of retinal arterioles, bone-spicule pigmentation in the midperiphery, atrophy of the retinal pigment epithelium, waxy-pale discs, and enlarged optic cups in the fundus of the proband (B) Normal fundus.

There were 8 affected indivisuals out of 24 members in this four-generation Chinese family and three of them were deceased. The deceased three patients displayed the similar symptoms of night blindness and progressive reduction of the visual field as the proband. The pedigree was consistent with autosomal dominant inheritance (Figure 1). The clinical data of the 5 living affected members are presented in Table 2. All affected individuals had visual disabilities but no history of systemic abnormalities. Night blindness was always the presenting symptom and the age of onset was between 10 to 15 years old. Affected members also reported a gradual decline in visual acuity and progressive peripheral visual field loss in their 40 s. Funduscopic examinations revealed similar clinical manifestations among affected individuals, including waxy-pale discs, attenuation of retinal arterioles, bone-spicule pigmentation in the midperiphery, and atrophy of the retinal pigment epithelium (Figure 2). Full field scotopic rod ERG showed either a reduced ERG response or non-recordable response.

**Table 2:** Clinical features of patients with the *SNRNP200* mutation.

	II:1		III:3		III:9		IV:6		IV:7	
	OD	OS	OD	OS	OD	OS	OD	OS	OD	OS
Age (years)/Sex	83/F		49/M		49/F		27/F		19/M	
Age of onset of night blindness (years)	10		12		10		14		12	
BCVA (logMAR)	NL	NL	0.1	0.2	0.2	NL	1.0	1.0	1.0	1.0
IOP(mmHg)	58	60	13	15	34	35	14	15	14	15
PSC	Yes	Yes	Yes	Yes	Yes	Yes	No	No	No	No
Optic disc	NA	NA	Pale	Pale	Pale	Pale	Cerinus	Cerinus	Cerinus	Cerinus
Artery attenuation	NA	NA	Yes	Yes	Yes	Yes	Yes	Yes	No	No
Pigment deposits	NA	NA	Int	Int	Int	Int	Diff	Diff	Diff	Diff
Visual field	NA	NA	30°	30°	10°	NA	60°	60°	30°	30°
ERG	NA	NA	NR	NR	NR	NR	Both remarkably reduced response		Both remarkably reduced response	

F: female; M: male; BCVA: best corrected visual acuity; logMAR: logarithm of the Minimum Angle of Resolution; NL: no light perception; IOP: intraocular pressure; PSC: posterior subcapsular cataract; ERG: electroretinography; NR: not recordable; Int: intensive Diff: diffuse; NA: not available.

Angle-closure glaucoma was diagnosed in two individuals (II:1 and III:9). Both patients were in their 40 s and complained of recurrent episodes of sudden onset of pain and decreased vision in both eyes. Ophthalmic examination of III:9 revealed a shallow anterior chamber and enlarged cup-to-disc ratio in both eyes (Figure 2). Anterior and posterior segment exams of II:1 could not be completed due to bullous keratopathy. The intraocular pressure of both patients were much higher than normal without any treatment.

### Exome Sequencing and Mutation Analysis

We sequenced the exome of the proband (III:9) and generated an average of 7.6 billion bases of sequence with a mean depth of target region of 87.5-fold. Approximately 99.33% (3862.72 Mb in length) of the targeted bases were covered sufficiently to pass our thresholds for calling SNPs and short insertions or deletions (Indels). The rate of nucleotide mismatch was below 0.3%. After identifying the variants, we focused only on non-synonymous (NS) variants, splice acceptor and donor site mutations (SS), and short, frame-shift coding insertions or deletions (Indels) that were more likely to be pathogenic than other variants.

**Figure 3:**
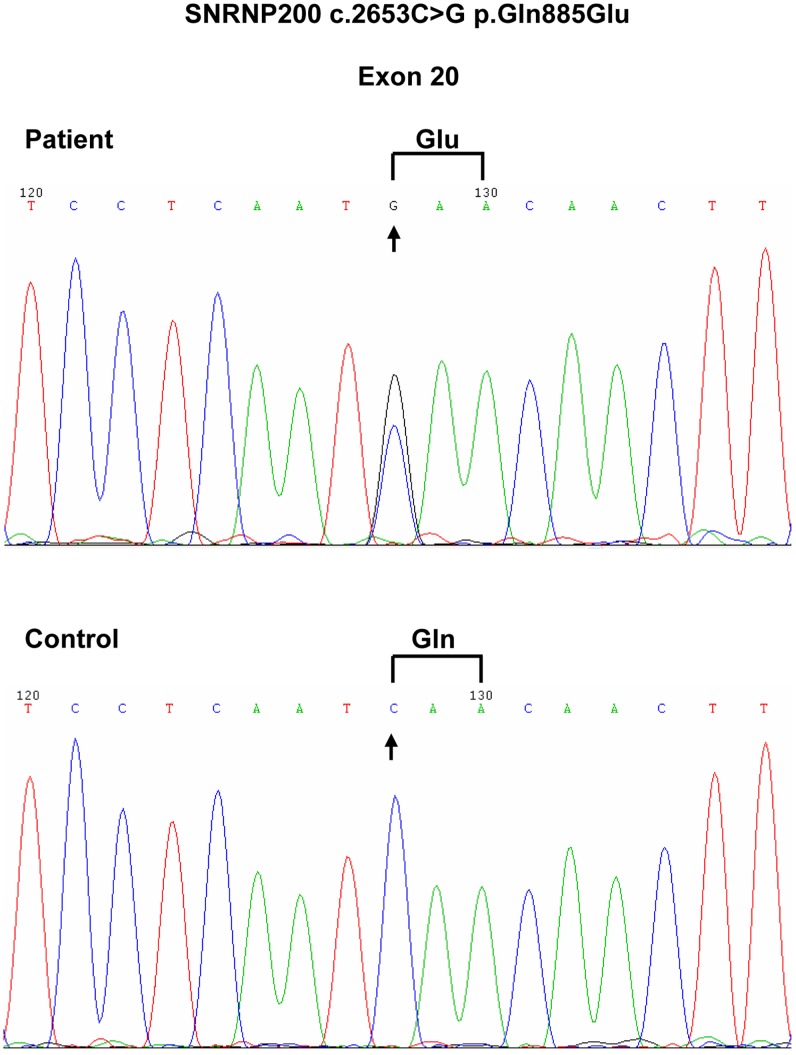
The *SNRNP200* sequencing results. (Top) Sequencing data shows that a C to G transversion (arrow) resulted in the substitution of glutamine-885 by glutamic acid (Q885E) in affected individuals. (Bottom) The corresponding normal sequence (arrow) was found in unaffected family members and controls.

In the proband, we identified 13,143 SNPs in the coding regions (12,894 missense, 88 readthrough and 161 nonsense), 3,083 variants in introns that may affect splicing (2,699 SNPs, 384 indels within 10 bp of the intron/exon junction), and 5,830 indels in coding regions or introns. Since RP is a rare disorder but has a clear phenotype, the probability of RP patients sharing casual mutations with the healthy population is very low. Therefore, we compared these variants in the proband with that of dbSNP132, HapMap project, 1000 Genome Project and YH database. After filtering against these databases, 1,642 variants (1,353 SNPs and 289 Indels) were left, out of which five mutations including *SNRNP200* c.2653C>G, *USH2A* c.997T>C, *USH2A* c.2750G>A, *USH2A* c.10246T>G and *PDE6B* c.1415G>A were not found in previously reported RP genes. Furthermore, these five mutations were selected for Sanger sequencing validation and segregation analysis on the available 14 members of the adRP family (Figure 1). *USH2A* c.997T>C and *USH2A* c.10246T>G were false heterozygous mutations, while *USH2A* c.2750G>A and *PDE6B* c.1415G>A were novel single nucleotide polymorphic sites. Only one variant co-segregated with the disease phenotype in this family: a C to G transversion in exon 20 (c.2653C>G), resulting in the Q885E mutation in *SNRNP200* (Figure 3). What’s more, this mutation was absent in 100 normal controls.

### Conservation of p.Q885

**Figure 4:**
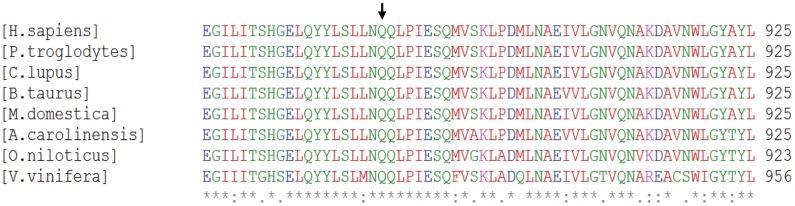
Multiple sequence alignment of *SNRNP200*. Sequence alignment of hBrr2 from eight species is shown. Q885E occurs at a highly conserved position in hBrr2 (arrow).

*SNRNP200* (also known as ASCC3L2) encodes hBrr2, which is one of the U5 snRNP-specific proteins (NP_054733.2) (named U5 snRNP-specific 200 kDa protein). The gene is located on chromosome 2q11.2. hBrr2 belongs to the DEXH-box family of putative RNA helicases [13]. We screened the SNRNP200 orthologs using the NCBI HomoloGene database (http://www.ncbi.nlm.nih.gov/sites/entrez?cmd=Retrieve&db=homologene&dopt=MultipleAlignment&list_uids=5859) and revealed that Q885 is highly conserved in eight species (Figure 4).

### Structure Modeling of p.Q885

**Figure 5:**
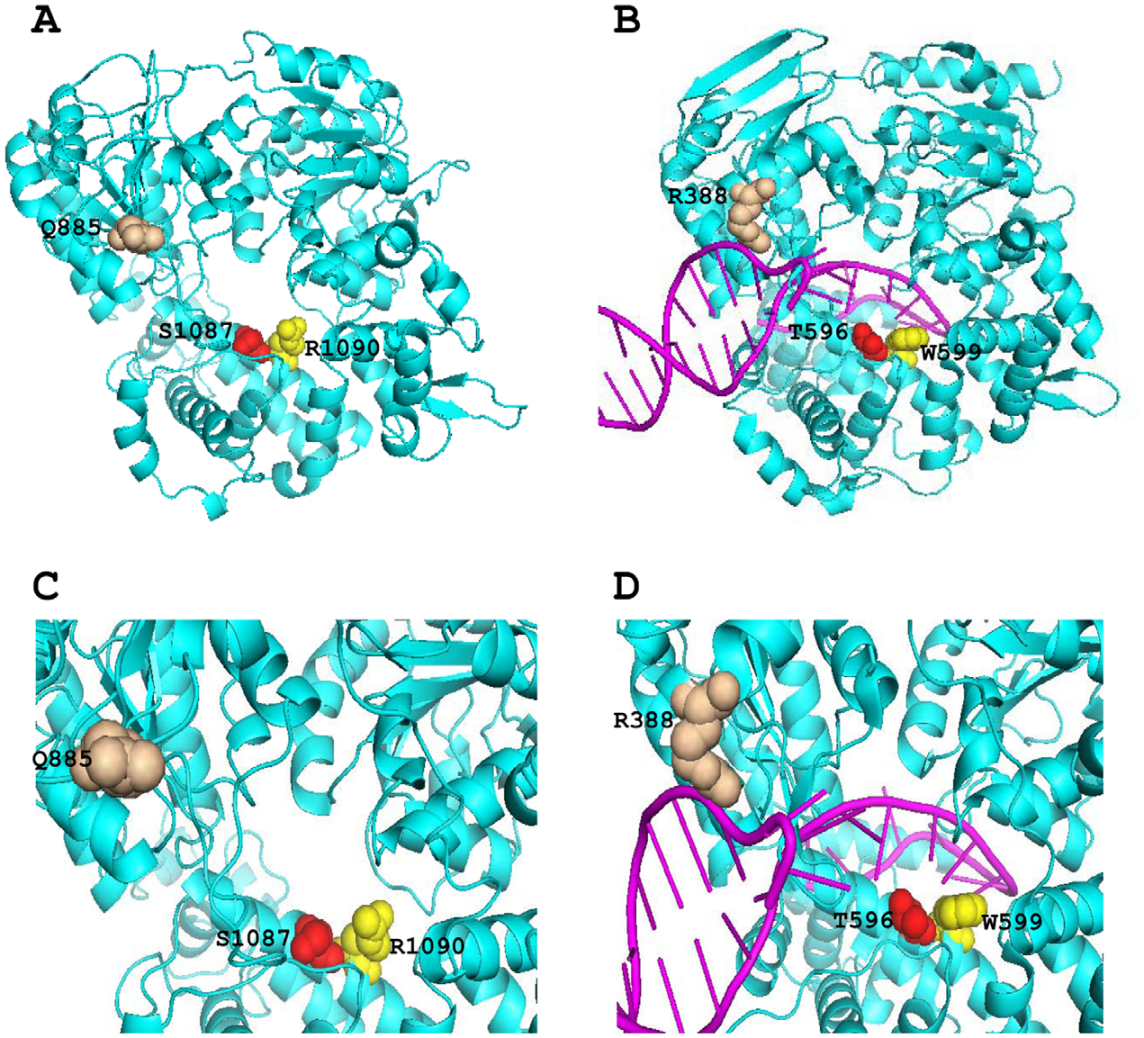
Structure modeling of hBrr2(477–1174). (A) Orthogonal ribbon plots of the model of hBrr2(477–1174). Three conserved residues (Q885, S1087 and R1090) that may interact with RNA base are shown as space-filling spheres, and colored as wheat, red and yellow respectively. (B) Orthogonal ribbon plots of the Hel308 DNA helicase [2] (PDB ID: 2p6r). Cyan, Hel308; pink, DNA. Three DNA interacting residues R388, T596 and W599 in Hel308 which correlate to Q885, S1087 and R1090 in human respectively are shown as space-filling spheres, and colored in order as wheat, red and yellow. (C) A close view of the central pore in the model, highlighting the three conserved residues (Q885, S1087 and R1090). (D) A close view of the path of DNA strand through the central pore, highlighting the three related residues (R388, T596 and W599) implicated in DNA binding.

To predict the functional changes of Q885E, we tried to model the structure of hBrr2 by using SWISS-MODEL server as a tool [14], and the structures were displayed by using PyMol software. A molecular model of hBrr2 was constructed based on the crystal structure of *Pfu*Hjm [15] (PDB ID: 2zj8), a DNA helicase which belongs to superfamily-2 helicase and shares homology with Hel308. The constructed model covered target sequence of hBrr2 (residues 477–1174), and was based on template PDB 2zj8. The sequence identity between the target and the template was 28%, a bit higher than average 25% sequence identity of many helicase superfamily members. From model it shows that hBrr2 (residues 477–1174) folds into five domains (RecA-like 1 and 2 domains, helical bundle, HLH and WH domains) [16] and possesses a hole at the center of molecule (Figure 5A), similar to that of Hel308-DNA complex [17] (Figure 5B). Three conserved amino acids across different species, highlighted as Q885, S1087 and R1090 of hBrr2 are shown as space-filling spheres (Figure 5A) in the model. In comparison, the 3 equivalent amino acids in Hel308, of which R388 equivalents to Q885 in human, T596 equivalents to S1087 in human and W599 equivalents to R1090 in human are shown as space-filling spheres (Figure 5B).

## Discussion

We identified a novel missense mutation (c.2653C>G p. Q885E) in *SNRNP200* associated with adRP in a Chinese family using a combination approach of exome sequencing and Sanger sequencing of candidate mutations. This novel mutation co-segregated with the RP phenotype over four generations and was absent in a panel of 100 normal controls.

*SNRNP200* encompasses 45 exons and encodes hBrr2, a 200-kDa helicase which is one of the U5 snRNP-specific proteins playing a critical role in pre-mRNA splicing process. Splicing is a enzymatic reactions takeing place in the spliceosome, a super molecular complex containing five small nuclear ribonucleoproteins (snRNPs) and ∼200 other proteins [18]. hBrr2 is a core component of U4/U6-U5 snRNPs and catalyzes unwinding of the U4/U6 snRNP duplex, which is a key step in the catalytic activation of the spliceosome [19], [20]. The protein is homologous to yest Brr2 and belongs to the DExD/H box protein family and possesses two consecutive Hel308-like modules (Hel308-1 and Hel308-2), each with a DExD/H box domain with ATPase activity and a Sec63 domain [16], [21], [22].

It is known that the first two adRP-assiociated mutations of hBrr2, p.S1087L and p.R1090L, are located at the α5 of the helical bundle in N-terminal of the first sec63 domain [16] involved in nucleic acid unwinding. Previous studies had elucidated that residues in Hel308, equivalent to hBrr2 residues S1087 and R1090, have direct contact with nucleic acids from Sec63 domain and are essential for duplex unwinding [16], [17], [22]. Here we found another adRP-associated mutation of hBrr2, p.Q885E and predicted the structure using in silicon analysis. Q885 falls into the Brr2 region containing the first DExD-helicase domain, which has been demonstrated to be essential for the U4/U6 unwinding function and critical for cell survival in yeast [20], [23]. In our structural model, Q885 in human is equivalent to R388 in Hel308, a key amino acid identified to have direct interaction with nucleic acids [17] (Figure 5A, 5B). Although the functional roles of Q885E mutation have not yet been elucidated by experiments, it is assumed that due to substitution of glutamine-885 to glutamic acid (Q885E), the electrostatic property of this niche area has been changed greatly by introduction of negatively charged side chain of glutamic acid, thus the Q885E mutation could influence the potential contact of its niche area to nucleic acids. Evidently, the first of the two Hel308-like modules shows the highest level of conservation among species, which reflects its importance at the functional level [22]. This is also consistent with the finding in yeast Brr2p, only the first Hel308-like repeat performs a catalytic role and the second repeat functions in protein binding and regulation by Prp8 [10]. This may explain why all the adRP mutations found are all located in the first DExD-helicase module.

Q885E may have serious implication for normal cellular processes. By disrupting U4/U6 unwinding and therefore stalling the splicesome disassembling, it may seriously compromise the efficiency of the global gene transcription and expression. This may be particularly critical in retinal cells, which are highly demanding in the accuracy and efficiency of opsin synthesis and active photoreceptors renewal. A defect in mRNA splicing could break a retinal cell cycle, yet would be tolerated in other tissues [10].

Interestingly, we found that there were some difference in the clinical features between mutation caused by *SNRNP200* p.Q885E in our study and the other two *SNRNP200* mutations (p.R1090L and p.S1087L) found in Chinese families. In our family, angle-closure glaucoma was also found in two adRP cases, which hasn’t been revealed in other reported Chinese families. RP has been associated with certain systemic and ocular findings, including open-angle glaucoma [24]–[25]. However, the association of angle-closure glaucoma with RP is rare in published reports, and it has even been proposed that this association may be fortuitous [26], Although none of the clinical and histopathological reports available for any type of glaucoma elucidates the pathogenesis of glaucoma in relation to RP, whether *SNRNP200* p.Q885E is associated angle-closure glaucoma calls for further attention.

In conclusion, we identified a new missense mutation c.2653C>G (p.Q885E) in exon 20 of *SNRNP200*, which is the seventh mutation site identified in *SNRNP200* that is associated with adRP. Our work expands the mutation spectrum of *SNRNP200* and contributes to elucidating the molecular pathogenesis of core spliceosomal components related to adRP.
